# Supplementary material for: Room-temperature super-elongation in high-entropy alloy nanopillars
Source: Nat Commun. 2023 Nov 17;14:7469. doi: 10.1038/s41467-023-42894-z (PMC10656519; doi:10.1038/s41467-023-42894-z)
Supplement: Supplementary file 1 — Supplementary Information [file 41467_2023_42894_MOESM1_ESM.pdf]

Supplementary Information

Room-temperature super-elongation in high-entropy alloy nanopillars

Qian Zhang[1,#], Ranming Niu[2,#], Ying Liu[2], Jiaxi Jiang[1], Fan Xu[3], Xuan Zhang[1], Julie M. Cairney[2], Xianghai An[2*], Xiaozhou Liao[2*], Huajian Gao[4,5*] & Xiaoyan Li[1*]

[1]Centre for Advanced Mechanics and Materials, Applied Mechanics Laboratory, Department of Engineering Mechanics, Tsinghua University, Beijing 100084, China

[2]School of Aerospace, Mechanical and Mechatronic Engineering, The University of Sydney, Sydney, NSW 2006, Australia

[3]Institute of Mechanics and Computational Engineering, Department of Aeronautics and Astronautics, Fudan University, Shanghai 200433, China

[4]School of Mechanical and Aerospace Engineering, College of Engineering, Nanyang Technological University, 70 Nanyang Drive, Singapore 639798, Singapore

[5]Institute of High Performance Computing, A*STAR, Singapore 138632, Singapore

[#]These authors contributed equally to this work

*e-mail: xianghai.an@sydney.edu.au; xiaozhou.liao@sydney.edu.au; huajian.gao@ntu.edu.sg; xiaoyanlithu@tsinghua.edu.cn

This file includes,

Supplementary Note 1

Supplementary Figures 1-13

Supplementary References 1-4

Supplementary Note 1. Partial dislocation nucleation from free surface.

Our molecular dynamics (MD) simulations in Supplementary Figs. 10a-c showed that partial dislocations nucleate from the corners of rectangular cross sections in samples[1], which initiates deformation twinning. Based on the energetics of twinning processes[2,3], an analytical model was developed to predict the critical stress for partial dislocation nucleation from the free surface with the configuration shown in Supplementary Fig. 10d. The dislocation line is simplified to be one quarter of a circle.

Driven by the resolved shear stress, the partial dislocation nucleates from the corner trailed by an intrinsic stacking fault. The free energy change ΔG during the nucleation process is associated with the dislocation line energy U_{line}, the change in stacking fault energy (SFE) U_{sf} and the work done by external force W_τ, i.e.,

$$\Delta G = U_{line} + U_{sf} - W_\tau \tag{1}$$

Substituting the specific expressions of U_{line}, U_{sf} and W_τ into Eq. (1), the free energy change is written as,

$$\Delta G = \int_{-\pi/4}^{\pi/4} \frac{Gb_p^2}{4\pi(1-\nu)}\left(1-\nu\cos^2\theta\right) R \ln\left(\frac{mR}{r_c}\right) d\varphi + \gamma_{sf}\cdot S(R) - \tau\cdot b_p\cdot S(R) \tag{2}$$

where G is the shear modulus, b_p the magnitude of Burgers vector of the partial dislocation, ν the Poisson ratio, R the dislocation loop radius, θ the angle between the Burgers vector and the dislocation line vector and equals to φ, r_c the dislocation core radius and is set to be b_p (Ref. 3), γ_{sf} the SFE, τ the resolved shear stress, $S(R)$ the area of stacking fault or the area swept by the partial dislocation, and m the line energy correction factor reflecting the effect of image stress from free surface. According to a previous theoretical study[2], the factor m is proportional to the wedge angle for corner nucleation, i.e.,

$$m = m_{BF}\left(\frac{\alpha}{\pi/2}\right) \tag{3}$$

where m_{BF} from the Beltz-Freund half-space solution[3] is equal to 0.55 for a Poisson's ratio of 0.3[3], and α is the half of the wedge angle equal to $\pi/4$ in the case of Supplementary Fig. 10d.

During nucleation, there exists a critical radius of dislocation loop[3], which is determined by $d(\Delta G)/dR = 0$. Once the radius of dislocation loop is beyond the critical value, the energy change becomes a monotonic decreasing function of the dissociation radius, meaning that the dislocation will expand spontaneously under the applied shear stress[3]. Note that $S(R)=\pi R^2/4$. Thus, let $d(\Delta G)/dR = 0$, we obtaiend the corresponding critical stress for partial dislocation nucleation as follows,

$$\tau_{crit} = \frac{Gb_p}{2R}\frac{1}{4\pi(1-\nu)}\left(1+\ln\frac{mR}{b_p}\right)\left(2-\nu-\frac{2\nu}{\pi}\right) + \frac{\gamma_{sf}}{b_p} \tag{4}$$

Previous experimental and theoretical studies[2,3] suggested that the critical radius of dislocation loop is generally comparable to the surface source size. For micro/nanopillars fabricated via focused ion beam (FIB), the surface source size is from several nanometers to ~10 nm[2,3]. Thus, the critical radius of dislocation loop in the experimental nanopillars is taken as about 5~10 nm. Substituting the material parameters (G=86 GPa, b_p=0.145 nm, ν=0.3, and γ_{sf}=30~34.8 mJ/m^2) for CoCrFeNi, m=0.275, and R=5~10 nm into Eq. (4), we obtained the critical twinning stress in the range of 628~903 MPa. Note that the tensile stress $\sigma = \tau_{\mathrm{crit}}/m_{\mathrm{Schmid}}$, where m_{Schmid} is the Schmid factor equal to 0.471 for twinning under <110>-oriented tension. Thus, the applied tensile stress for the corner nucleation of partial dislocations is estimated between 1.33 to 1.92 GPa, which is in line with the yield strength range in our experiments as shown in Fig. 1e. It should be noted that the strength of high entropy alloy (HEA) nanopillars from our MD simulation is as high as 4~6 GPa. Such high strength is related to smaller pillar size, smaller critical dislocation radius and much higher strain rate than the experiments.

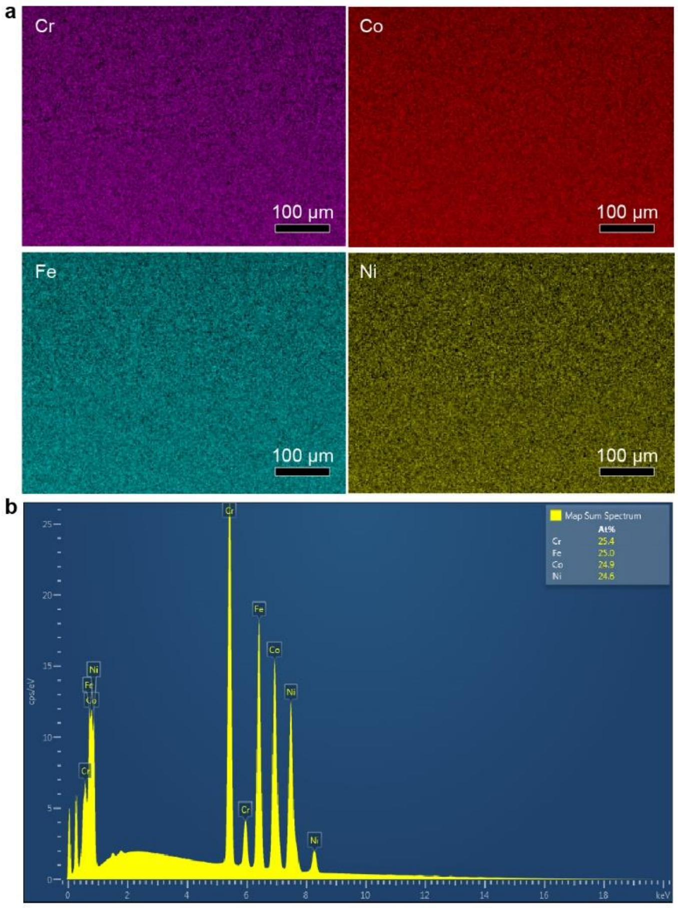

Supplementary Fig. 1 | Energy dispersive X-Ray spectroscopy of the well-annealed CoCrFeNi HEA. a, Elemental maps of Cr, Co, Fe and Ni, showing homogeneous distribution of the principal elements in the well-annealed sample. **b,** An energy dispersive X-Ray spectrum from the sample area in **a**, showing near equiatomic compositions of the CrCoFeNi HEA.

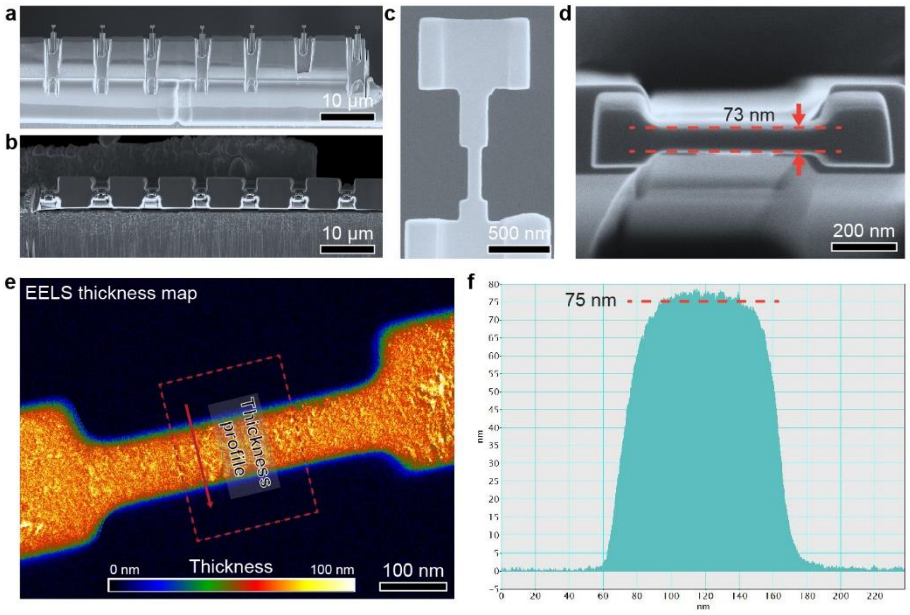

Supplementary Fig. 2 | Nanopillars with a dog-bone shape and well-defined geometry for quantitative in-situ tensile TEM testing. a, b, The scanning electron microscopy (SEM) images of an array of nano-dog-bone samples from the surface-on and edge-on directions. The samples were fabricated on a piece of single crystalline lamellar that was lifted out from a single grain with specific orientation, which was determined using electron backscatter diffraction (EBSD), in the HEA by using focused ion-beam and micro-manipulator. **c,** An SEM image of a single-crystalline HEA nanopillar from the surface-on direction. **d,** An edge-on view of the same sample shows the thickness was ~73 nm. **e,** Thickness mapping with electron energy loss spectroscopy suggests a high uniformity in thickness of the sample. **f.** Averaged line profile at the marked area in **e** shows the thickness of the sample was ~75nm, which is almost identical to the SEM measurement results in **d**.

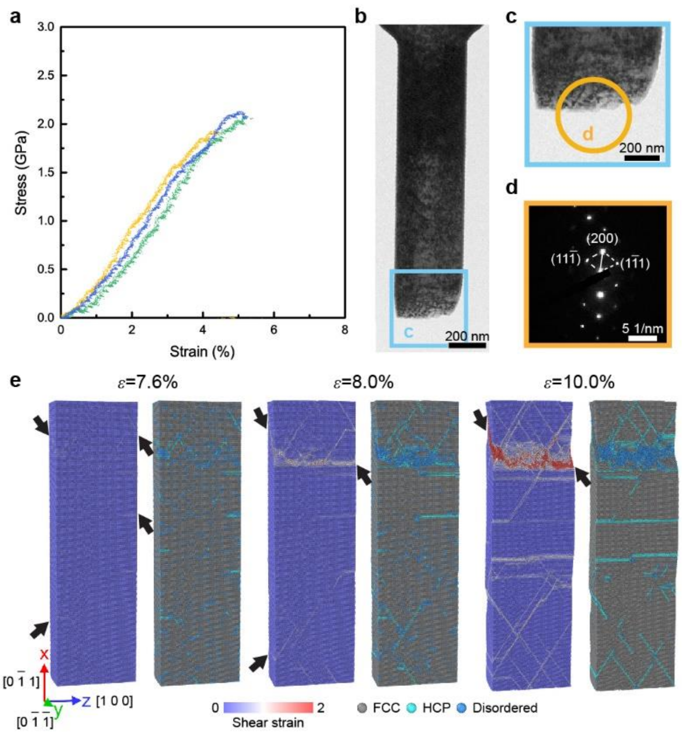

Supplementary Fig. 3 | Uniaxial tension of the single crystalline <100>-oriented HEA nanopillars. a, Tensile engineering stress-strain curves of <100>-oriented nanopillars. **b,** TEM image of <100>-oriented nanopillar after failure. **c,** The close-up TEM image of fracture site in **b**, indicating a brittle fracture mode. **d,** The selected-area electron diffraction (SAED) pattern of fracture surface in **c**. **e,** A sequence of snapshots of simulated nanopillar under stretching, indicating that dislocation slip dominates the plastic deformation leading to fracture. The black arrows in **e** indicate the dislocation activities.

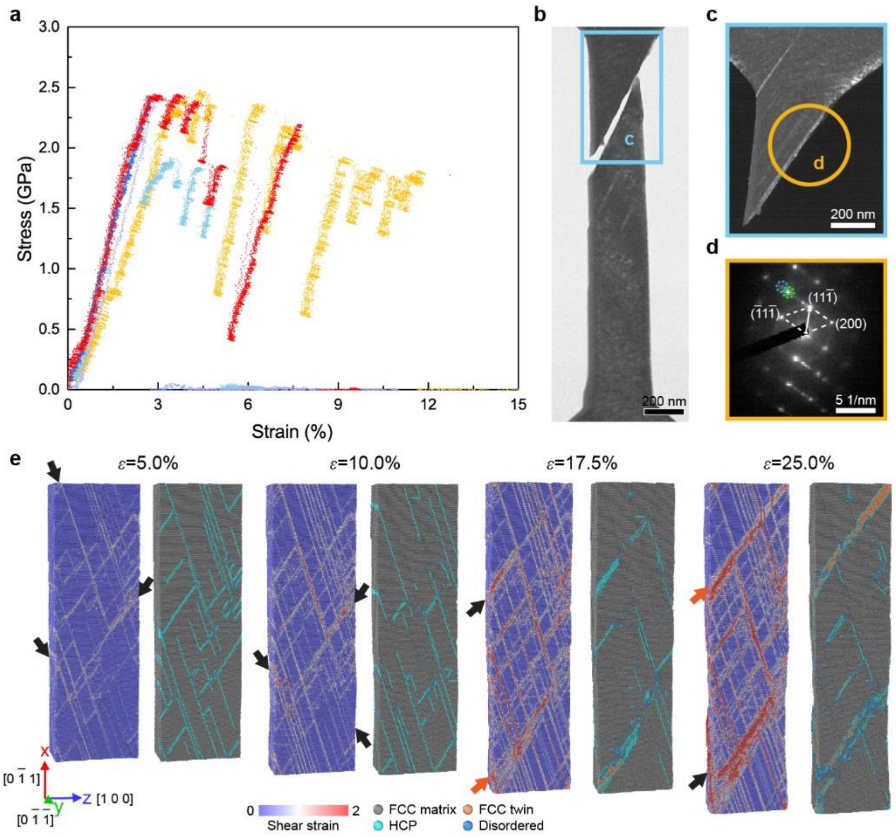

Supplementary Fig. 4 | Uniaxial tension of the single crystalline <111>-oriented HEA nanopillars. a, Tensile engineering stress-strain curves of <111>-oriented nanopillars. **b,** TEM image of <111>-oriented nanopillar after failure. **c,** The dark field TEM image of fracture site in **b**, suggesting failure by shear localization from deformation twinning. **d,** The SAED pattern of fracture surface in **c**. **e,** A sequence of snapshots of simulated nanopillar under stretching, indicating that dislocation slip within a thin twin leads to the premature failure. The black and orange arrows indicate dislocation slip and deformation twinning, respectively.

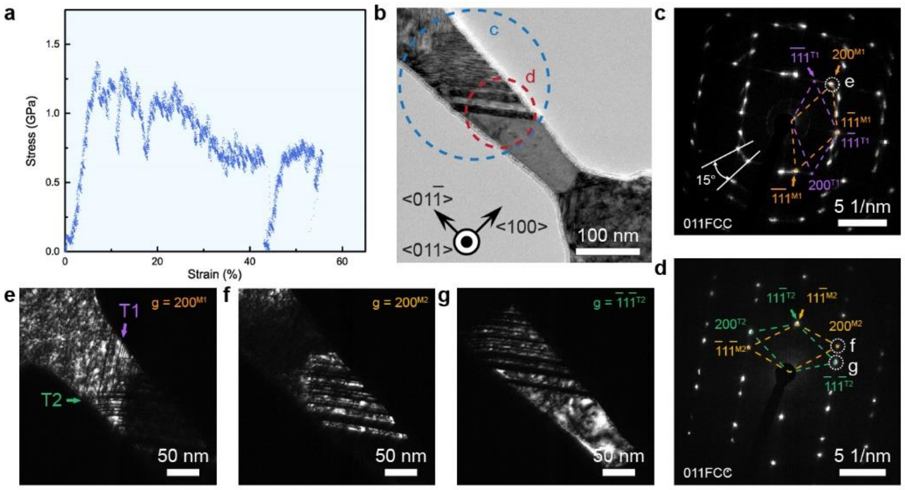

Supplementary Fig. 5 | Post-mortem TEM results of a <110>-oriented HEA nanopillar. a, Tensile engineering stress–strain curve of the sample. The *in-situ* tensile testing was deliberately stopped at the strain of 55.3% for a post-mortem study of typical deformation events that are responsible for the super-elongation have been spatially activated. **b,** The bright-field TEM image shows the morphology of the deformed sample. Note that an amorphous layer was grown by carbon contamination during the post-mortem TEM observations. The original thickness before the post-mortem TEM (and during *in-situ* testing) was 2~5 nm (See Fig. 2), which is normal in samples prepared by FIB. **c, d,** The SAED patterns collected at marked areas in **b**. The diffraction pattern in **c** contains four sets of patterns corresponding to the original matrix (M1, orange), deformation twinning on $(1\bar{1}1)$ planes (Twin 1, purple), reoriented matrix (M2, yellow), and deformation twinning on $(11\bar{1})$ planes (Twin 2, green)**.** The pattern in **d** collected at the center of the sample using a small selected-area aperture identifies M2 and Twin 2, showing $[200]^{T2}$ orientation aligned upwards with the sample loading axis. **e-g,** The dark-field TEM images, with *g* vectors applied to 200^{M1}, 200^{M2} and $\bar{1}1\bar{1}^{T2}$, in turns reveal M1, M2 and T2 as bright contrast, respectively. The positions of the objective aperture when taking the dark-field images are marked by white broken circles in **c** and **d**. The dark-field images demonstrate extensive twinning activities in the sample. A sub-grain boundary was detected in **e** at where Twin 1 and Twin 2 actively interacted, leading to the reorientation from M1 to M2 at the middle part of the sample. In **f,** a vast of Twin 2 were seen lying in M2 with increasing thickness along the loading axis to the lower part of the sample. A region with a bright contrast in **b** was fully twinned due to the migration and coalescences of Twin 2, which can also be confirmed in the dark field image in **g**.

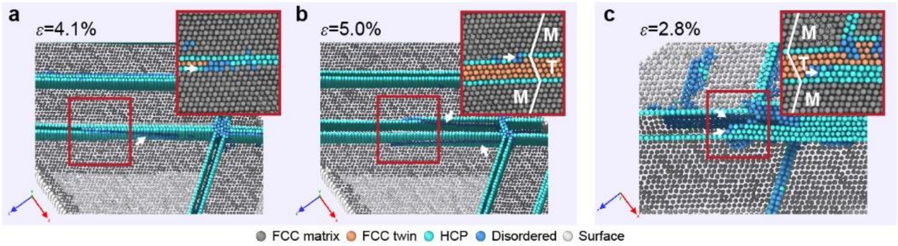

Supplementary Fig. 6 | Nucleation and growth of nanotwins in the <110>-oriented HEA nanopillars during tension. a, b, Nucleation and growth of deformation twins due to successive slip of dislocations. **c,** Formation of deformation twin due to interaction between two SFs and dislocations. The white arrows indicate the motion of partial dislocations.

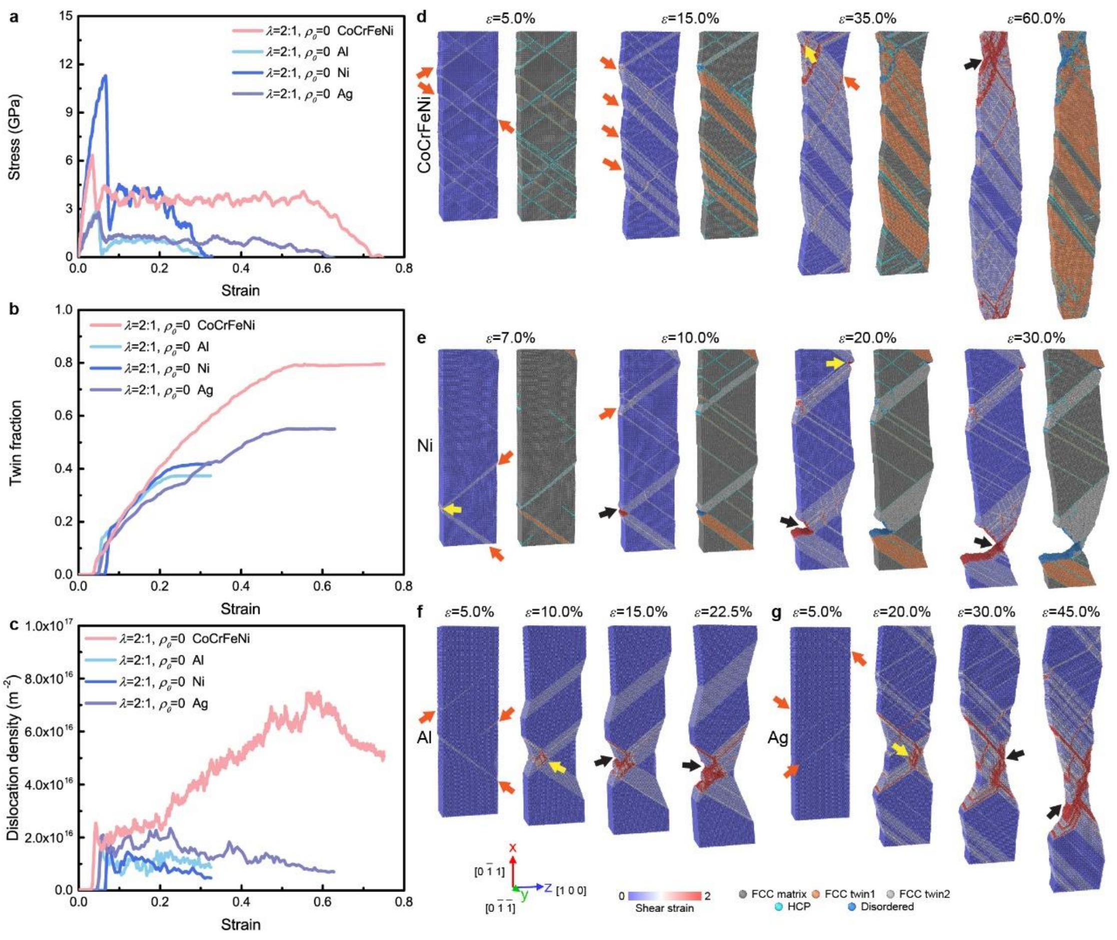

Supplementary Fig. 7 | Comparison of tensile behaviors between single crystalline <110>-oriented CoCrFeNi HEA and pure metallic (Al, Ni and Ag) nanopillars. a, Simulated tensile stress-strain curves of the HEA and pure metallic nanopillars. **b, c,** Simulated evolutions of twin fraction and dislocation density with the tensile strain in HEA and pure metallic nanopillars, respectively. **d,** A sequence of snapshots of the HEA nanopillar without initial dislocation during tension, indicating that plastic deformation is dominated by deformation twinning, leading to a large uniform elongation of the HEA nanopillar at room temperature. **e,** A sequence of snapshots of the stretched Ni nanopillar, suggesting that although plastic deformation is dominated by both dislocation slip and deformation twinning, dislocation slip induces deformation localization, resulting in early failure of the Ni nanopillar at a tensile strain of ~30%. **f, g,** A sequence of snapshots of the stretched Al and Ag nanopillars. It indicates the occurrence of deformation localization induced by twin-twin interaction. Due to the low SFE of Ag, relatively significant dislocation slip and twinning are activated, resulting in the moderate twin fraction and elongation of Ag nanopillars. The dislocation slip and deformation twinning in **d**, **e**, **f** and **g** are indicated by black and orange arrows, respectively. The twin-twin interactions are indicated by the yellow arrows in **d**, **e**, **f** and **g**.

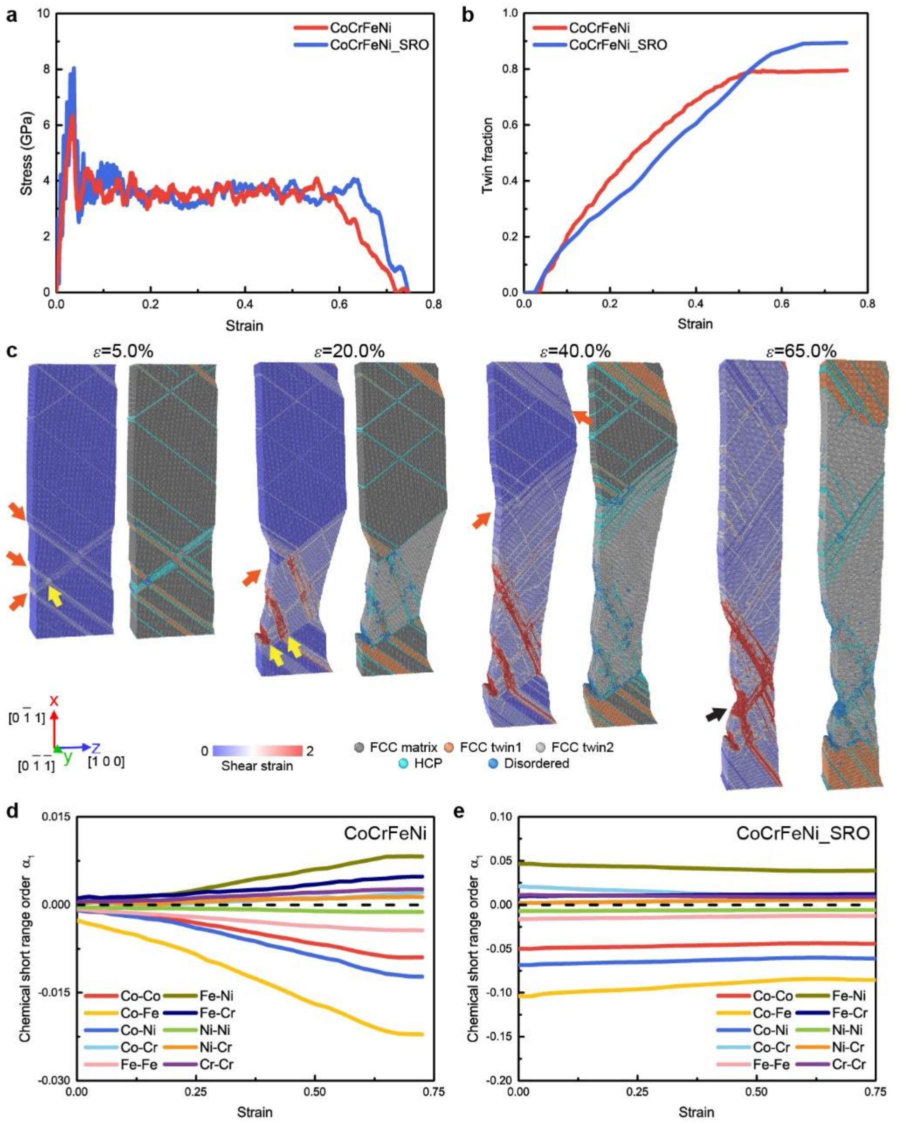

Supplementary Fig. 8 | Atomistic simulations of <110>-oriented HEA nanopillars without and with shor-range orders (SROs) under uniaxial tension. a, Tensile stress-strain curves of the HEA nanopillars without and with SRO. **b,** Effect of the SRO on the evolution of twin fraction with the tensile strain in HEA nanopillars with different SRO extents. **c,** A sequence of snapshots of HEA nanopillar with SRO during tension. The orange and black arrows indicate the deformation twinning and dislocation slip, respectively. The yellow arrows indicate the interactions between two twins. **d,** Variation of the chemical SRO parameter with the tensile strain in simulated HEA nanopillar without SRO. **e,** Variation of the chemical SRO parameter with the tensile strain in simulated nanopillars with SRO.

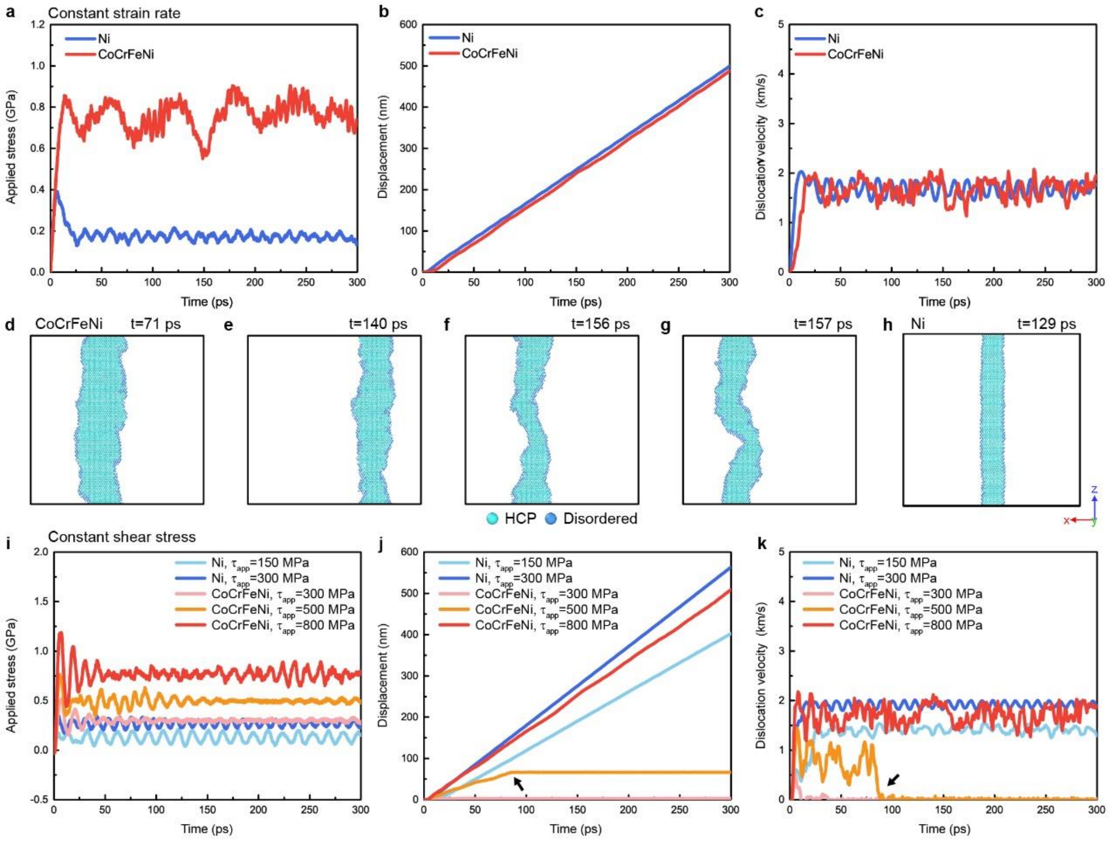

Supplementary Fig. 9 | The 1/2<110>{111} dislocation mobility in the single-crystalline HEA and Ni samples. a, Evolution of applied stress for edge dislocation motion in HEA and Ni under the same strain rate. **b,** Displacements of edge dislocaitons in HEA and Ni under the same strain rate. **c,** Velocities of edge dislocations in HEA and Ni under the same constant strain rate. **d-g,** A sequence of snapshots of sliding of an extended dislocation (i.e. an SF ribbon bounded by two partial dislocations) in HEA sample. These snapshots indicate wavy configurations of a dislocation during motion due to significant lattice distortion. **h,** A snapshot of sliding of an extended dislocation with straight disocation lines and a uniform SF width in the Ni sample. **i,** Evolution of applied stress for edge dislocation motion in HEA and Ni under constant shear stress. **j,** Displacements of edge dislocations in HEA and Ni under constant shear stress. **k,** Velocities of edge dislocations in HEA and Ni under constant shear stress. The black arrows in **j** and **k** indicate that dislocation in HEA can be pinned even after moving for a certain distance due to varied local energy barrier originated from chemical compositional heterogeneities at the atomic level.

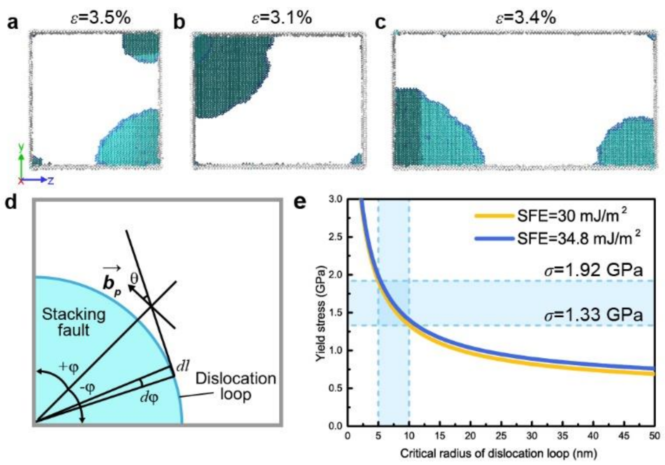

Supplementary Fig. 10 | Calculation of critical stress for a heterogenous nucleation of partial dislocations in HEA nanopillars. a-c, Partial dislocations nucleation from corners of samples with different cross-sections from our MD simulations. **d,** Schematic illustration of partial dislocation nucleation from a corner on free surface. **e,** Variation of the yield stress with the critical radius of dislocaiton loop. The SFE values of 30 mJ/m^2 and 34.8 mJ/m^2 are the experimentally measured value and the average calculated value based on the 5,000 generalized planar fault energy (GPFE) curves, respectively.

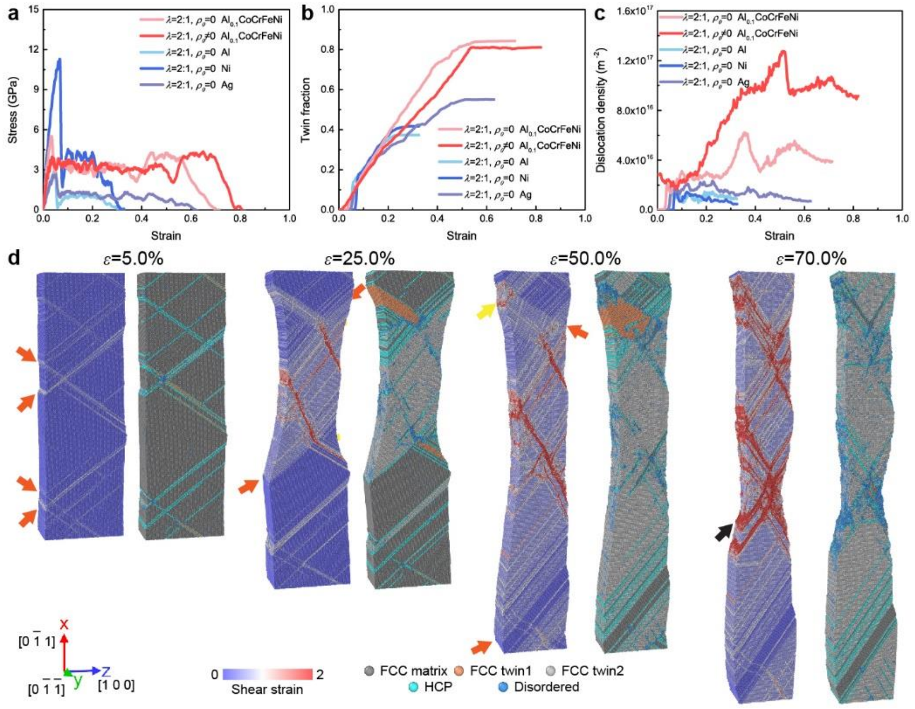

Supplementary Fig. 11 | Atomistic simulations of <110>-oriented $Al_{0.1}$CoCrFeNi HEA nanopillars under uniaxial tension. a, Tensile stress-strain curves of simulated HEA nanopillars. **b, c,** Simulated evolutions of twin fraction and dislocation density with the tensile strain in HEA nanopillars, respectively. The stress-strain curve and evolutions of twin fraction and dislocation density of simulated nanopillars for pure metals (Al, Ni, and Ag) are included in **a-c** for comparison. **d,** A sequence of snapshots of a stretched nanopillar with λ=2:1 and $\rho_0 \neq 0$. The atoms are colored according to their von Mises shear strains and local crystalline structures, respectively. The orange arrows and black arrows indicate the deformation twinning and dislocation slip, respectively. The yellow arrows indicate the twin-twin interaction.

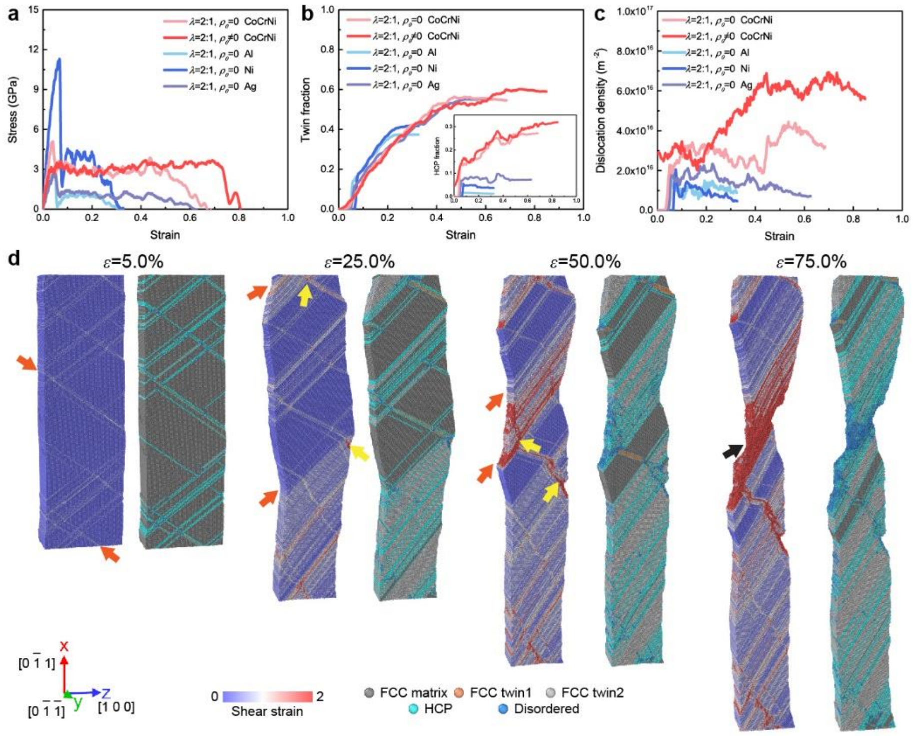

Supplementary Fig. 12 | Atomistic simulations of <110>-oriented CoCrNi medium entropy alloy (MEA) nanopillars under uniaxial tension. a, Tensile stress-strain curves of simulated MEA nanopillars. **b, c,** Simulated evolutions of twin fraction and dislocation density with the tensile strain in MEA nanopillars, respectively. The inset in **b** indicates the fraction of atoms with hexagonal close-packed (HCP) structure. The stress-strain curve and evolutions of twin fraction, HCP fraction, and dislocation density of simulated nanopillars for pure metals (Al, Ni, and Ag) are included in **a-c** for comparison. **d,** A sequence of snapshots of a stretched nanopillar with λ=2:1 and $\rho_0 \neq 0$. The atoms are colored according to their von Mises shear strains and local crystalline structures, respectively. The orange arrows and black arrows indicate the deformation twinning and dislocation slip, respectively. The yellow arrows indicate the twin-twin interaction.

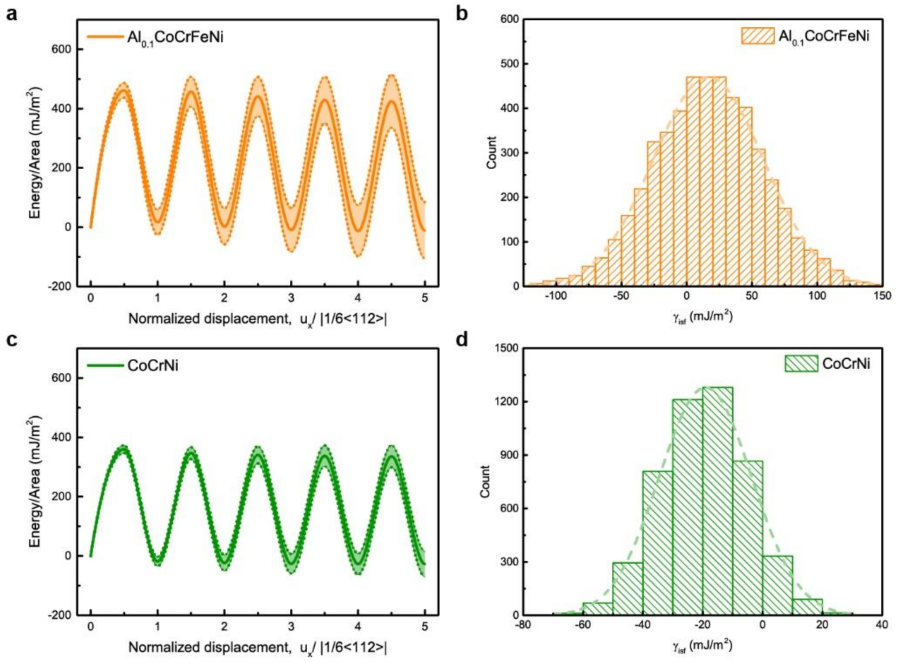

Supplementary Fig. 13 | Calculation of GPFE curves for HEAs. a, c, GPFE curves of $Al_{0.1}CoCrFeNi$ and CoCrNi samples, respectively. The curves are taken as the average from the GPFE curves of 5,000 samples with different random solid solutions. The shadows show the deviation of such a statistical average. **b, d,** Statistical distributions of intrinsic SFE of $Al_{0.1}CoCrFeNi$ HEA and CoCrNi MEA from 5,000 calculations.

Supplemental References

1 Weinberger, C. R., Jennings, A. T., Kang, K. & Greer, J. R. Atomistic simulations and continuum modeling of dislocation nucleation and strength in gold nanowires. *J. Mech. Phys. Solids* **60**, 84-103 (2012).

2 Jennings, A. T. *et al.* Modeling dislocation nucleation strengths in pristine metallic nanowires under experimental conditions. *Acta Mater.* **61**, 2244-2259 (2013).

3 Liang, Z. & Huang, M. Deformation twinning in small-sized face-centred cubic single crystals: Experiments and modelling. *J. Mech. Phys. Solids* **85**, 128-142 (2015).

4 Beltz, G. E. & Freund, L. B. On the Nucleation of dislocations at a crystal-surface. *Phys. Status Solidi B* **180**, 303-313 (1993).
