# Supplementary material for: Room-temperature super-elongation in high-entropy alloy nanopillars
Source: Nat Commun. 2023 Nov 17;14:7469. doi: 10.1038/s41467-023-42894-z (PMC10656519; doi:10.1038/s41467-023-42894-z)
Supplement: Supplementary file 3 — Description of Additional Supplementary Files [file 41467_2023_42894_MOESM3_ESM.pdf]

Description of Additional Supplementary Files

File Name: Supplementary Movie 1

Description: In-situ transmission electron microscopy (TEM) tensile testing of a <110>-oriented high entropy alloy nanopillar (playing speed x10). The scale bar is 100 nm.

File Name: Supplementary Movie 2

Description: Atomistic simulation for uniaxial tension of a simulated <110>-oriented nanopillar with λ=2 and ρ_0>0.
